# ZMIZ2/MCM3 Axis Participates in Triple-Negative Breast Cancer Progression

**DOI:** 10.32604/or.2025.066662

**Published:** 2025-12-30

**Authors:** Xiaopan Zou, Meiyang Sun, Xin Jiang, Jingze Yu, Xiaomeng Li, Bingyu Nie

**Affiliations:** 1 Breast and Thyroid Surgery, Jilin Province People’s Hospital, Changchun, 130021, China; 2 The Key Laboratory of Molecular Epigenetic, Institute of Genetics and Cytology, Northeast Normal University, Changchun, 130024, China; 3 Plastic Surgery Department, Shenzhen Art Star Medical Cosmetology Hospital, Shenzhen, 518000, China; 4 KingMed School of Laboratory Medicine, Guangzhou Medical University, Guangzhou, 510182, China

**Keywords:** Triple-negative breast cancer, zinc finger miz-type containing 2, minichromosome maintenance complex component 3, pathway enrichment analysis

## Abstract

**Objective:**

Triple-negative breast cancer (TNBC) is highly aggressive and lacks an effective targeted therapy. This study aimed to elucidate the functions and possible mechanisms of action of zinc finger miz-type containing 2 (ZMIZ2) and minichromosome maintenance complex component 3 (MCM3) in TNBC progression.

**Methods:**

The relationship between ZMIZ2 expression and clinical characteristics of TNBC was investigated. *In vitro* and *in vivo* experiments were performed to investigate the role of ZMIZ2 dysregulation in TNBC cell malignant behaviors. The regulatory relationship between ZMIZ2 and MCM3 was also explored. Transcriptome sequencing was performed to elucidate possible mechanisms underlying the ZMIZ2/MCM3 axis in TNBC.

**Results:**

High ZMIZ2 expression levels were associated with the malignant degree of TNBC. ZMIZ2 overexpression promoted TNBC cell proliferation, migration, and invasion; inhibited apoptosis; and induced G1 phase cell cycle arrest, whereas knockdown of ZMIZ2 had the opposite effect. ZMIZ2 directly targeted and positively regulated MCM3 expression. MCM3 knockdown reversed the effect of ZMIZ2 overexpression on TNBC tumor growth both *in vitro* and *in vivo*. High MCM3 expression levels were linked to the degree of malignancy and poor prognosis in TNBC. The differentially expressed genes associated with the ZMIZ2/MCM3 axis were significantly enriched in multiple pathways, such as the mitogen-activated protein kinase (MAPK), mechanistic target of rapamycin (mTOR), Wnt, and Ras signaling pathways, as verified by The Cancer Genome Atlas data.

**Conclusions:**

ZMIZ2 and MCM3 were highly expressed in TNBC. ZMIZ2 promoted the development by positively regulating MCM3 expression. Key pathways, such as the Ras/MAPK, phosphatidylinositol 3-kinase (PI3K)/protein kinase B (AKT)/mTOR, and Wnt signaling pathways, may be key downstream mechanisms.

## 1 Introduction

Breast cancer is a common malignant tumor caused by environmental, genetic, and hormonal factors [1]. Triple-negative breast cancer (TNBC) is an aggressive breast cancer subtype that represents 15%–20% of all breast cancers [2]. This subtype often occurs in young women, 40% of whom die within 5 years of diagnosis [3]. Approximately 50% of the patients with advanced TNBC develop distal metastasis to the brain and/or internal organs [4]. High rates of distal metastasis, together with frequent recurrence and poor survival, contribute to the aggressive nature of TNBC [5,6]. Considering these factors and the lack of effective therapies, TNBC is frequently associated with poor clinical outcomes. Approximately 30%–40% of patients with early-stage TNBC develop metastatic disease and eventually succumb to the disease despite receiving multi-agent adjuvant chemotherapy [7]. Therefore, elucidating TNBC progression and identifying potential molecular therapeutic targets are imperative.

Accurate and complete DNA replication is crucial for tumor cell proliferation. Minichromosome maintenance (MCM) proteins, comprising six members (MCM2–7), are crucial players in DNA replication and cell cycle progression [8]. In late mitosis and the G1 phase, MCM2–7 complexes are loaded onto DNA to form the core of the eukaryotic replicative helicase, which is essential for unwinding duplex DNA and initiating fork progression to enable DNA replication [9,10]. Additionally, MCM complexes can be loaded onto chromatin at the beginning of S phase. Simultaneously, it performs the recombinational repairs of stalled or collapsed replication forks upon activation [11,12]. MCM complex component 3 (MCM3), a subunit of the MCM2–7 protein complex, is essential for coordinating cell proliferation and differentiation [13]. MCM3 is activated in most cancers [14]. Elevated MCM3 expression has been observed in multiple cancers, such as papillary thyroid carcinoma [15], hepatocellular carcinoma [16], and endometrial carcinoma [17]. MCM3 is a proliferation marker in invasive ductal breast cancer [18]. However, the role of MCM3 in TNBC remains largely unknown.

The zinc finger miz-type containing 2 (ZMIZ2), also known as hZIMP7, is a member of the protein inhibitor of activated STAT (PIAS) protein family that interacts with nuclear hormone receptors and enhances androgen receptor-mediated transcription [19]. ZMIZ2 expression is elevated in human colorectal cancer (CRC) tissues and is associated with poor clinical outcomes [20]. Our previous study showed the upregulation of ZMIZ2 in both TNBC tissues and cell lines [21]. Although our previous study has revealed a potential relationship between ZMIZ2 and MCM3 [21], whether ZMIZ2 promotes TNBC via MCM3 and the underlying regulatory mechanisms remains unclear.

Several signaling pathways regulate tumor growth and metastasis. The RAS-mitogen-activated protein kinase (MAPK) pathway acts as a central hub that processes external signals and regulates cell growth, survival, and differentiation [22]. This pathway is implicated in breast cancer invasion and metastasis [23,24], and is linked to a favorable prognosis in node-positive TNBC [25]. In addition, other pathways, such as the phosphatidylinositol 3-kinase (PI3K)/protein kinase B (AKT)/mechanistic target of rapamycin (mTOR), Ras/MAPK, and Wnt/β-catenin pathways, contribute to TNBC development [26]. However, the signaling pathways that mediate the roles of ZMIZ2 and MCM3 in TNBC development remain unclear.

This study investigated the functions of ZMIZ2 and MCM3 in TNBC both *in vivo* and *in vitro*. We also determined the MCM3 expression in clinical TNBC samples and analyzed its prognostic value. In addition, transcriptome sequencing was performed to elucidate the regulatory pathways of the ZMIZ2/MCM3 axis in TNBC, which were verified using The Cancer Genome Atlas (TCGA) TNBC data analysis and experiments. The purpose of this study was to elucidate the functions and possible mechanisms of action of ZMIZ2 and MCM3 in TNBC progression. Our findings will reveal the regulatory mechanisms underlying TNBC and provide potential targets for the treatment of this aggressive breast cancer subtype.

## 2 Materials and Methods

### 2.1 Clinical Samples

Twenty-four tissue samples (six luminal breast cancer, six HER2+ breast cancer, six TNBC, and six adjacent normal tissues) were collected from Jilin Province People’s Hospital. The patient did not undergo radiotherapy or chemotherapy. All patients provided informed consent, and the study was approved by the Institutional Ethics Committee of Jilin Province People’s Hospital (2023129).

### 2.2 Cell Culture

Two TNBC cell lines, MDA-MB-231 and MDA-MB-468, and the normal breast epithelial cell line, MCF-10A, were purchased from Procell Life Science & Technology Co., Ltd. (Wuhan, China). They were cultured in Leibovitz’s L-15 complete medium with 10% extra-grade fetal bovine serum (Procell Life Science & Technology Co., Ltd., Wuhan, China) at 37°C in a cell incubator. MDA-MB-231 and MDA-MB-468 cells were authenticated by short tandem repeat (STR) analysis (Shanghai Biowing Applied Biotechnology, Shanghai, China) and regularly tested for *Mycoplasma* contamination by polymerase chain reaction (PCR).

### 2.3 Plasmid Construction and Transfection

To study the functional roles of ZMIZ2 and MCM3 in TNBC, shRNAs targeting ZMIZ2 (ZMIZ2-sh, 5^′^-TGCTGTTGACAGTGAGCGACCAGCTGCGAGACTCAGTCTATAGTGAAGCCACAGATGTATAGACTGAGTCTCGCAGCTGGATGCCTACTGCCTCGGA-3^′^) and MCM3 (MCM3-sh, 5^′^-GCATGACTATGTCAAGAAAGC-3^′^) were designed and synthesized for the knockdown of ZMIZ2 and MCM3. An shRNA non-target sequence (5^′^-GTTCTCCGAACGTGTCACGT-3^′^) was used as a negative control. For ZMIZ2 overexpression, the ZMIZ2 sequence (ZMIZ2-OE) was integrated into a lentiviral plasmid (Genewiz Biotechnology Co., Ltd., Suzhou, China). To generate high-titer lentiviruses, the lentiviral vectors were co-transfected into MDA-MB-231 and MDA-MB-468 cells with packaging plasmids using the Lipofectamine 2000 transfection kit (Cat. No. 11668019; Invitrogen; Thermo Fisher Scientific, Inc., Waltham, MA, USA). At 48 h post-transfection, cells were harvested for further experiments.

### 2.4 RNA Isolation and Quantitative Reverse Transcription PCR (qRT-PCR)

Total RNA was isolated from ZMIZ2-OE-transfected MDA-MB-231 cells using the TRIzol reagent (Cat. No. 15596018CN; Invitrogen, USA). After reverse transcription, gene expression was detected by qRT-PCR using SYBR Green qPCR Master Mix (Servicebio, Beijing, China). PCR systems were 2× TB Green Premix Ex Taq 10 μL, forward primer (100 ng/μL) 0.5 μL, reverse primer (100 ng/μL) 0.5 μL, and cDNA 2 μL, added with dH_2_O to 20 μL. Cycling parameters were as follows: initial denaturation at 95°C for 3 min; followed by 40 cycles of denaturation at 95°C for 15 s and annealing at 60°C for 30 s. Glyceraldehyde-3-phosphate dehydrogenase (GAPDH) was used as the internal control. Relative quantification was performed using the 2^–ΔΔCT^ method. The primers used were as follows:

MCM3-F: 5^′^-GCGCAGGAAAAACGAGAAGAG-3^′^,

MCM3-R: 5^′^-AATGGAGGCCACAAAATCCTTT-3^′^;

ZMIZ2-F: 5^′^-CTTCTTGCCTGATCTCAAGCC-3^′^,

ZMIZ2-R: 5^′^-GGAAGACATGGTTGCTTACAGC-3^′^;

GAPDH-F: 5^′^-GGAGCGAGATCCCTCCAAAAT-3^′^,

GAPDH-R: 5^′^-GGCTGTTGTCATACTTCTCATGG-3^′^.

### 2.5 Cell Counting Kit 8 (CCK-8) Assay

MDA-MB-231 and MDA-MB-468 cells without treatment and those transfected with ZMIZ2-sh, ZMIZ-OE or their controls (1000 cells per well) were seeded into 96-well plates, followed by the addition of 10 μL of a CCK8 reagent (Cat. No. E606335; Sangon Biotech, Shanghai, China) to each well. The plates were then incubated for 1 h at 37°C in the dark. The absorbance values at 450 nm were measured using a microplate reader (Epoch2, MultiskanGo, Thermo Fisher Scientific, Vantaa, Finland).

### 2.6 Cell Apoptosis Assay

MDA-MB-231 and MDA-MB-468 cells without treatment and those transfected with ZMIZ2-sh, ZMIZ-OE or their controls were digested by 0.25% trypsin and collected for apoptosis detection using an Annexin V-FITC/PI Apoptosis Detection Kit (Cat. No. 40302ES20; Yeasen, Shanghai, China). Briefly, the cells (1 × 10^6^ cells/mL) were stained with Annexin V-FITC and propidium iodide (PI) for 15 min in the dark. A flow cytometer (FC500; Beckman Coulter, Fullerton, CA, USA) was used to detect apoptosis after 1 h.

### 2.7 Transwell Assays

Briefly, MDA-MB-231 and MDA-MB-468 cells without treatment and those transfected with ZMIZ2-sh, ZMIZ-OE or their controls were added to a Transwell insert (Corning, NY, USA), and medium containing 20% fetal bovine serum (Gibco, Thermo Fisher Scientific, Waltham, MA, USA) was placed on the other side of the insert. After conventional culture for 24 h and fixation with 4% formaldehyde for 30 min, the Transwell chamber was stained with 0.1% crystal violet (BBI Life Sciences, Shanghai, China) for 30 min. Cell migration and invasion were observed under a fluorescence microscope (CKX41; Olympus, Tokyo, Japan). Unlike in the migration assay, the insert was pre-coated with Matrigel (BD Biosciences, Franklin Lakes, NJ, USA) for the invasion assay.

### 2.8 Cell Cycle Assay

After collection, MDA-MB-231 and MDA-MB-468 cells without treatment and those transfected with ZMIZ2-sh, ZMIZ-OE or their controls were fixed overnight with 75% cold ethanol. Cells were then incubated with RNase at 37°C for 30 min and stained with 0.1 mg/mL of PI for 10 min. Finally, flow cytometry (FC500; Beckman Coulter, Fullerton, CA, USA) was used to analyze the cell cycle.

### 2.9 Chromatin Immunoprecipitation Assay (ChIP)

MDA-MB-231 cells were lysed with 400 μL of 1% sodium dodecyl sulfate (SDS) lysate on ice for 10 min. After centrifugation, the supernatant was divided into two portions (475 μL each): one was treated with the ZMIZ2 antibody (2 μg, 1:200, Cat. No. PA5-59263; Invitrogen; Thermo Fisher Scientific, Inc., Waltham, MA, USA) and the other with the same amount of IgG (1:200; Cat. No. GB25303; Servicebio, Beijing, China) as a negative control. Samples were then incubated overnight at 4°C and washed six times afterwards as follows: low salt once, high salt twice, lithium chloride once, and Tris-EDTA (10 mM Tris and 1 mM EDTA, pH 8.0) twice. They were added with 200 μL elute buffer (0.1 M NaHCO_3_, 1% SDS) for precipitation using magnetic beads. Finally, the expression was quantified using real-time PCR. Cycling parameters were as follows: initial denaturation at 94°C for 2 min; followed by 30 cycles of denaturation at 94°C for 30 s, annealing at 50°C for 30 s, and extension at 72°C for 30 s. The primer sequences (5^′^ to 3^′^) were as follows:

MCM3 promoter-F1: CCCACACAAATAGATGGCCT

MCM3 promoter-R1: TACTGGATTGCCCAACATCAG

MCM3 promoter-F2: AAAAAGCCAACCACGACGGA

MCM3 promoter-R2: AACGATCAGCTCAAGAGGGG

MCM3 promoter-F3: CAATGCTTTCCCTCTCGCCA

MCM3 promoter-R3: ACTGACGAGCAAACTGACCA

MCM3 promoter-F4: CAGTCGCTAGTCCGACCTC

MCM3 promoter-R4: GGCGCGAAAACTTCCGAAC

### 2.10 Immunofluorescence Assay

MDA-MB-231 and MDA-MB-468 cells transfected with ZMIZ-OE or its controls were fixed with pure methanol for 30 min and incubated with immunostaining blocking buffer (Beyotime, Shanghai, China) for 30 min. Cells were probed with ZMIZ2 (1:400, Cat. No. PA5-59263; Invitrogen; Thermo Fisher Scientific, Inc., Waltham, MA, USA) and MCM3 antibodies (1:400, Cat. No. PA5-29106; Invitrogen; Thermo Fisher Scientific, Inc., Waltham, MA, USA) overnight at 4°C, respectively. FITC (Servicebio, Beijing, China) and CY3 (Servicebio, Beijing, China) labeled with fluorescent goat anti-rabbit IgG secondary antibodies (1:400 dilution; Cat. No. GB25303; Servicebio, Beijing, China) were then added to incubate cells at 37°C for 1 h. Nuclei were stained with 3 μg/mL 4^′^,6-diamidino-2-phenylindole for 15 min. The images were captured using a fluorescence microscope (CX41; Olympus, Tokyo, Japan).

### 2.11 Dual-Luciferase Reporter Assay

ZMIZ2-OE and empty plasmids were transfected into cells using the Lipofectamine 2000 transfection kit (Cat. No. 11668019; Invitrogen; Thermo Fisher Scientific, Inc., Waltham, MA, USA). After 48 h, cell lysates were collected, and the luciferase activities of fireflies at the MCM3 promoter and Renilla were measured using a Dual-Luciferase Reporter System (E1910, Promega Corp., Madison, WI, USA).

### 2.12 Western Blotting

To detect the effect of ZMIZ2/MCM3 axis on the protein expression of ZMIZ2, MCM3, epithelial–mesenchymal transition-markers (E-cadherin and N-cadherin), apoptosis-related markers (BCL2 and BAX), cell cycle-related marker (MCM3 and cyclinA), and RAS/MAPK pathway-related markers (ERK1/2 and p-ERK1/2), western blot was conducted. An appropriate amount (5 μg) of MDA-MB-231 and MDA-MB-468 cells without treatment and those transfected with ZMIZ2-sh, ZMIZ-OE or their controls were lysed with radioimmunoprecipitation assay lysis buffer (Cat. No. P0013B; Beyotime, Shanghai, China) was added to lyse the on ice for 30 min. Total protein was quantified using a bicinchoninic acid protein detection kit (Cat. No. P0010; Beyotime, Shanghai, China) and separated using 10% SDS-PAGE and transferred to a polyvinylidene fluoride membrane (Millipore Co., Billerica, MA, USA). The PVDF membrane was then blocked with the Tris-buffered saline with Tween 20 (TBST) containing 5% skim milk at room temperature on a shaker for 1 h. The primary antibodies to ZMIZ2 (1:1000, Cat. No. PA5-59263; Invitrogen; Thermo Fisher Scientific, Inc., Waltham, MA, USA), MCM3 (1:1000, Cat. No. PA5-29106; Invitrogen; Thermo Fisher Scientific, Inc., Waltham, MA, USA), E-cadherin (1:1000, Cat. No. 20874-1-AP; Proteintech, Rosemont, IL, USA), N-cadherin (1:1000, Cat. No. 22018-1-AP; Proteintech, Rosemont, IL, USA), BCL2 (1:1000; Cat. No. MA5-11757; Invitrogen; Thermo Fisher Scientific, Inc., Waltham, MA, USA), BAX (1:2000; Cat. No. 50599-2-lg; Proteintech, Rosemont, IL, USA), cyclinA (1:1000; Cat. No. ab181591; Abcam, Cambridge, MA), ERK1/2 (1:1000, Cat. No. 61-7400; Invitrogen; Thermo Fisher Scientific, Inc., Waltham, MA, USA), p-ERK1/2 (1:1000, Cat. No. 44-680G; Invitrogen; Thermo Fisher Scientific, Inc., Waltham, MA, USA), and GAPDH (1:1000, Cat. No. PA1-16777; Invitrogen; Thermo Fisher Scientific, Inc., Waltham, MA, USA) were added to incubate membranes at 4°C overnight. After TBST (pH 7.4) washing, a goat anti-rabbit lgG secondary antibody (1:5000; #7074; CST, Beverly, MA, USA) was added and incubated for 1 h at 37°C with shaking. The protein signals were revealed using ECL detection solution (Cat. No. NCI5079; Thermo Fisher Scientific, Inc., Waltham, MA, USA). Protein expression was determined by analysis of gray values using the ImageJ software (version 1.41o, National Institutes of Health, Bethesda, MD, USA).

### 2.13 Xenograft Experiment

Female BALB/c nude mice aged 6–7 weeks (Hangzhou Ziyuan Laboratory Animal Technology Co., Ltd., Zhejiang, Jilin, China) were acclimatized for 1 week prior to the experiment. Mice were randomly assigned into four groups: control, MCM3-sh, ZMIZ2-OE, and ZMIZ2-OE+MCM3-sh groups (n = 3 in each group). For tumor implantation, MDA-MB-231 cells transfected with the empty plasmid, MCM3-sh, ZMIZ2-OE, or ZMIZ2-OE+MCM3-sh in the logarithmic phase were mixed with Matrigel. Then, 0.1 mL of the suspension containing 2 × 10^6^ cells was subcutaneously injected into the groin of each mouse. Tumor growth was recorded twice weekly, with tumor size measurements using calipers. Tumor volume was calculated using the equation: volume = (length × width²)/2, where length and width are measured in millimeters. After 3 weeks, the mice were sacrificed. The tumors were collected, weighed, cut into pieces, and stored. Protein expression was detected in the tumors using western blotting and immunohistochemistry. Animal experiments were approved by the Animal Experimental Ethics Committee of Changchun University of Chinese Medicine (2024156).

### 2.14 Hematoxylin–Eosin (HE) Staining

The tumor tissues of xenograft mouse models were fixed and embedded in paraffin. The 4-μm paraffin sections were subjected to hematoxylin-eosin (HE) staining (Cat. No. G1003, Servicebio, Beijing, China) for 5 min at room temperature (25°C). Pathological changes in the different tissues were observed under a light microscope (CX41; Olympus, Tokyo, Japan).

### 2.15 Immunohistochemistry (IHC)

The 4-μm paraffin sections of tumor tissues of xenograft mouse models were incubated with antibodies to Ki67 (Cat. No. GB111499; 1:400; Servicebio, Beijing, China) and IgG secondary antibodies (1:400 dilution; Cat. No. GB25303; Servicebio, Beijing, China). Ki67 expression was calculated using the Image-Pro Plus software (version 6.0; Media Cybernetics Corporation, Silver Spring, MD, USA).

### 2.16 Transcriptome Sequencing and Analysis

The total RNA was extracted from tumor tissues of xenograft mouse models in the control, MCM3-sh, and ZMIZ2-OE groups. Total RNA was then incubated with oligo (dT) magnetic beads. The cDNA library was established using an NEBNext^®^ Ultra™ RNA Library Prep Kit for Illumina^®^ (NEB, Ipswich, MA, USA). Subsequently, sequencing was performed on an Illumina HiSeq 6000 sequencing platform (Illumina, Inc., San Diego, CA, USA), and more than 6 G of raw data were obtained for each sample.

The clean reads obtained were aligned to the reference genome (GRCh38) using the Hisat2 (v2.1.0) software. The number of reads was determined using HTSeq v0.6.1 [27]. The FPKM of each gene was then computed. Differentially expressed genes (DEGs) in the ZMIZ2-OE vs. control, MCM3-sh vs. control, and ZMIZ2-OE vs. MCM3-sh groups were identified using the ‘DESeq2’ package (v4.0.4) [28] in R (version 3.6.1). The selection threshold was an adjusted *p-*value (adjusted by Benjamini and Hochberg’s method) < 0.05 and |log_2_ fold change (FC)| > 1. The DEGs were visualized using a Volcano plot. Kyoto Encyclopedia of Genes and Genomes (KEGG) pathways that were significantly enriched by DEGs were analyzed using the R ‘clusterProfiler’ package (version 3.14.3) [29].

### 2.17 Analysis of Key Regulatory Pathways Associated with TNBC Based on The Cancer Genome Atlas (TCGA) Data

From TCGA database (https://cancergenome.nih.gov/, accessed on 01 April 2025), the RNA-seq and clinical data for 122 TNBC samples and 113 normal samples were acquired. The DEGs in the TNBC and normal samples were calculated using the R ‘limma’ package (version 3.38.3) [30]. The thresholds were |log FC| > 0.75 and adj. *p*-value < 0.05. Gene Set Enrichment Analysis (GSEA) was performed to enrich the pathways in the TNBC and normal samples. The cutoff values were |normalized enrichment score (NES)| > 1.5 and *p* < 0.05. Moreover, the KEGG pathways significantly enriched by the DEGs were explored using the R ‘clusterprofile’ package.

### 2.18 Expression Analysis of RAS/MAPK Pathway-Related Genes

To explore the effect of the ZMIZ2/MCM3 axis on the RAS/MAPK pathway, we determined the expression of RAS/MAPK pathway-related markers (Ras, ERK, MEK1, and Raf) *in vitro* and *in vivo* by qRT-PCR and western blotting, as described above.

### 2.19 Statistical Analysis

All experiments were repeated three times. The data are expressed as means ± standard deviations and were compared using a one-way analysis of variance. Qualitative clinical data between the groups were compared using the chi-square (χ^2^) test. Kaplan–Meier survival analysis was conducted to evaluate the prognostic value of MCM3, followed by a log-rank statistical test. Statistical analysis was performed using the SPSS software (version 25.0; SPSS Inc., Chicago, IL, USA). Statistical significance was set at *p* < 0.05.

## 3 Results

### 3.1 High ZMIZ2 Expression Level Correlated with the Malignant Degree of TNBC

In our previous study, ZMIZ2 was remarkably upregulated in TNBC, and high ZMIZ2 expression levels were linked to poor prognosis in patients with TNBC [21]. We examined the association between ZMIZ2 expression and the clinical characteristics of TNBC. Patients with tumor diameter <2.0 cm exhibited a significantly lower ZMIZ2 expression level than those with tumor diameter >2.0 cm (*p* < 0.001; Table 1). Moreover, ZMIZ2 expression increased with increasing tumor–node–metastasis (TNM) stage (*p* = 0.002). However, ZMIZ2 expression did not differ significantly according to the histological grade or lymph node metastasis.

**Table 1:** The ZMIZ2 expression in TNBC patients with different clinical parameters

Clinical features	N	ZMIZ2 expression				χ^2^	*p*
		–	+	++	+++		
Tumor size						23.090	<0.001
≤2 cm	66	31	15	16	4		
>2 cm	76	11	23	10	30		
Histological grade						9.784	0.134
1	44	6	12	10	16		
2	42	17	11	6	8		
3	56	19	15	10	12		
Lymph node metastasis						5.022	0.170
N = 0	50	12	19	8	11		
N > 0	92	30	19	18	25		
TNM stage						14.945	0.002
I-IIb	51	24	14	7	6		
IIIa-IV	91	18	24	19	30		

Note: ZMIZ2, zinc finger miz-type containing 2; The expression intensity of immunostaining was graded as: –, negative; +, weak; ++, moderate; and +++,strong.

### 3.2 ZMIZ2 Overexpression Promotes the Malignant Behaviors of TNBC Cells, Whereas Knockdown of ZMIZ2 Has the Opposite Effect

**Figure 1:**
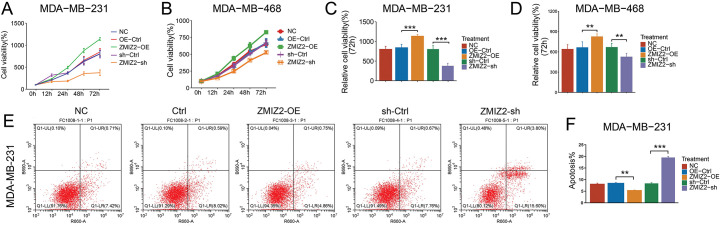

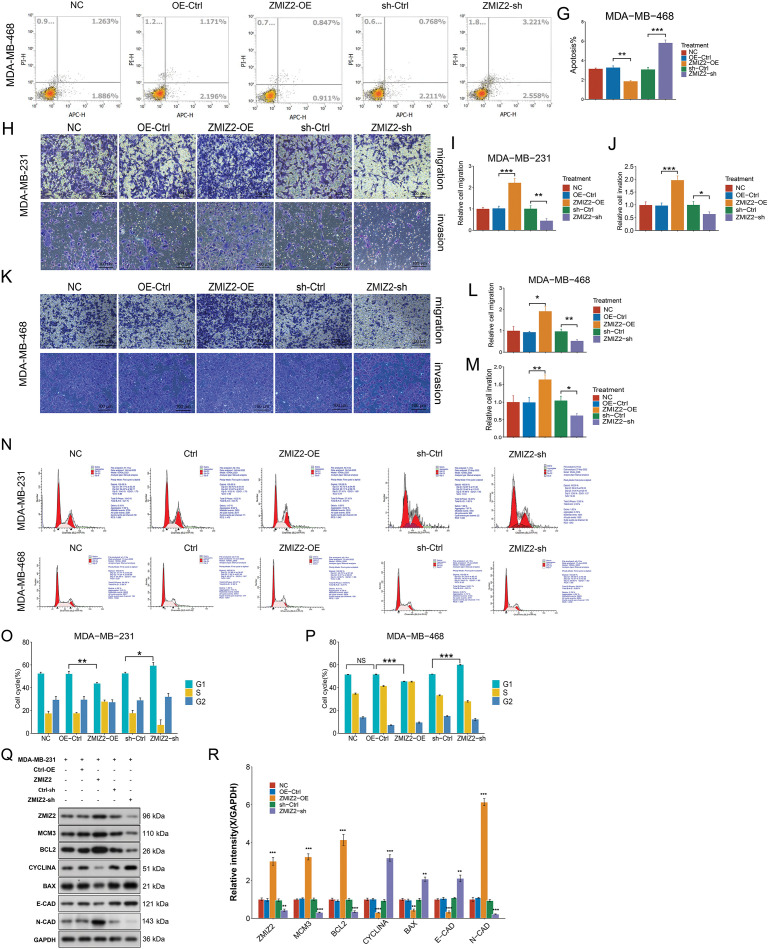
Zinc finger miz-type containing 2 (ZMIZ2) overexpression promotes the malignant behaviors of TNBC cells. (**A**–**D**): Cell Counting Kit-8 assay showing the viability of MDA-MB-231 and MDA-MB-468 cells after 72 h of transfection. (**E**–**G**): Flow cytometry revealed the effect of ZMIZ2 overexpression or knockdown on the apoptosis of MDA-MB-231 and MDA-MB-468 cells. (**H**–**M**): Transwell assays showing the effect of ZMIZ2 overexpression or knockdown on the migration and invasion of MDA-MB-231 and MDA-MB-468 cells. (**N**–**P**): Flow cytometry analysis of the effect of ZMIZ2 overexpression or knockdown on the cell cycle of MDA-MB-231 and MDA-MB-468 cells. (**Q**,**R**): Western blotting was used to detect the effects of ZMIZ2 overexpression and knockdown on the expression of epithelial–mesenchymal transition-, apoptosis-, and cell cycle-related markers in MDA-MB-231cells. NS, Not Significant, *p < 0.05, **p < 0.01, and ***p < 0.001

To analyze the biological function of ZMIZ2 in TNBC cells, we successfully overexpressed or knocked down ZMIZ2 in TNBC cells by transfection with ZMIZ2-OE and ZMIZ2-sh, respectively. The viability of MDA-MB-231 cells and MDA-MB-468 cells in the ZMIZ2-OE group was higher than that of the control group (Fig. 1A–D), whereas cell viability was decreased after transfection with ZMIZ2-sh (Fig. 1B,D). Flow cytometry analysis demonstrated that the apoptotic percentages of MDA-MB-231 and MDA-MB-468 cells markedly decreased after transfection with ZMIZ2. By contrast, apoptosis in MDA-MB-231 and MDA-MB-468 cells was dramatically increased after ZMIZ2 knockdown (Fig. 1E–G). Transwell assays showed that ZMIZ2 overexpression increased the migration and invasion of MDA-MB-231 and MDA-MB-468 cells, whereas ZMIZ2 knockdown had the opposite effect (Fig. 1H–M). Cell cycle analysis showed that ZMIZ2 overexpression significantly decreased the proportion of MDA-MB-231 and MDA-MB-468 cells in the G1 phase, indicating that the cell cycle was arrested in the G1 phase, whereas ZMIZ2 knockdown significantly increased the proportion of MDA-MB-231 and MDA-MB-468 cells in the G1 phase (Fig. 1N–P). Additionally, we detected the effects of ZMIZ2 dysregulation on the expression of epithelial–mesenchymal transition (EMT)-related proteins (N-cadherin and E-cadherin), apoptosis-related regulators (BCL2 and BAX), and cell cycle-related markers (MCM3 and cyclin A) in MDA-MB-231cells. BCL2, N-cadherin, and MCM3 protein expressions were significantly upregulated in the ZMIZ2-OE group, whereas BAX, E-cadherin, and cyclin A were significantly downregulated (Fig. 1Q,R). ZMIZ2 knockdown had the opposite effects on the expression of these proteins (Fig. 1Q,R). Overall, ZMIZ2 overexpression promoted the malignant behavior of TNBC cells by promoting cell proliferation, migration, and invasion, inhibiting apoptosis, and inducing G1 phase cell cycle arrest.

### 3.3 ZMIZ2 Interacts with and Positively Regulates MCM3

**Figure 2:**
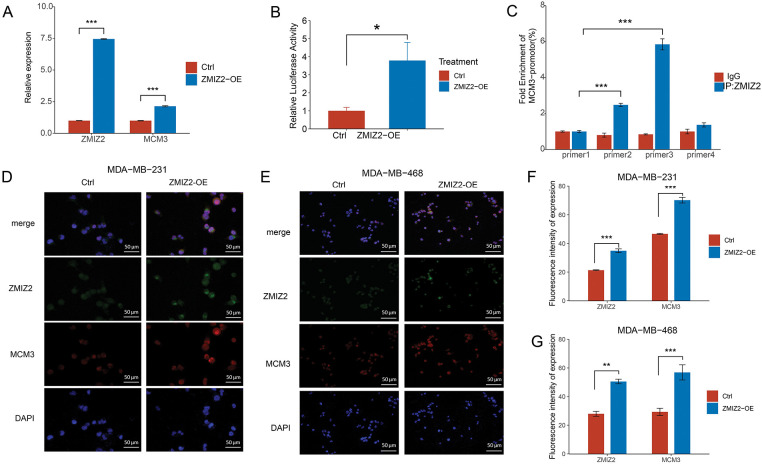
ZMIZ2 can directly interact with and positively regulate minichromosome maintenance complex component 3 (MCM3). (**A**): Quantitative reverse transcription polymerase chain reaction showing the MCM3 mRNA expression after ZMIZ2 overexpression. (**B**): Dual luciferase reporter assay showing the interaction between ZMIZ2 and MCM3. (**C**): ChIP assay determined the interaction between ZMIZ2 and MCM3. (**D**–**G**): Immunofluorescence assay showing the expression of MCM3 protein in MDA-MB-231 and MDA-MB-468 cells after ZMIZ2 overexpression. *p < 0.05, **p < 0.01, ***p < 0.001

In our previous study, ZMIZ2 interacted with MCM3 in TNBC samples [21]. Therefore, we further characterized the relationship between ZMIZ2 and MCM3. MCM3 mRNA expression was elevated in MDA-MB-231 cells after ZMIZ2 overexpression (Fig. 2A). Additionally, ZMIZ2-overexpressed MDA-MB-231 cells showed increased dual-luciferase activity of the MCM3 promoter, suggesting that ZMIZ2 directly targeted MCM3 in TNBC cells (Fig. 2B). Next, we clarified whether ZMIZ2 could bind to a specific site in the MCM3 promoter using a ChIP assay. ZMIZ2 overexpression promoted the binding of ZMIZ2 to P2 and P3 fragments, especially in P3 (Fig. 2C). Immunofluorescence assays indicated that MCM3 protein expression dramatically increased in MDA-MB-231 and MDA-MB-468 cells after ZMIZ2 overexpression (Fig. 2D–G), suggesting a positive correlation between ZMIZ2 and MCM3. Overall, these results implied that ZMIZ2 could interact with and positively regulate MCM3 in TNBC cells.

### 3.4 MCM3 Was Upregulated in TNBC and High MCM3 Expression Levels Correlated with TNBC Malignant Degree and Poor Prognosis

**Figure 3:**
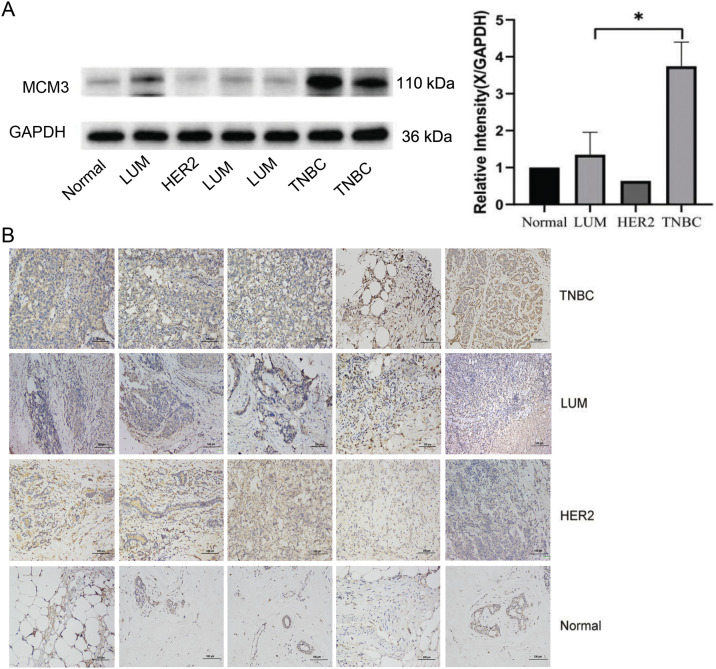

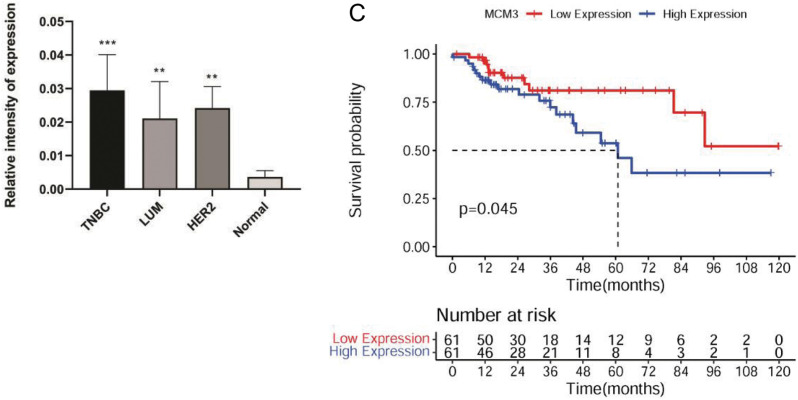
MCM3 expression in clinical breast cancer tissues and its prognostic value in patients with TNBC. (**A**): Western blotting showing MCM3 protein expression in normal, luminal breast cancer, HER2+ breast cancer, and TNBC tissues. (**B**): IHC assay revealing MCM3 protein expressions in normal, luminal breast cancer, HER2+ breast cancer, and TNBC tissues. (**C**): Kaplan–Meier survival curve showing the prognostic value of MCM3 in patients with TNBC. *p < 0.05, **p < 0.01, and ***p < 0.001

To further confirm the relationship between ZMIZ2 and MCM3, we examined the association between MCM3 expression and the clinical characteristics of TNBC as well as the correlation between ZMIZ2 and MCM3 expression in clinical samples. MCM3 protein expression was dramatically increased in TNBC samples (*p* < 0.05; Fig. 3A). IHC assays revealed that MCM3 protein was remarkably upregulated in luminal breast cancer, HER2+ breast cancer, and TNBC tissues (*p* < 0.01, Fig. 3B), and MCM3 expression was highest in TNBC tissues. Analyzing the clinical characteristics of TNBC revealed a positive MCM3 expression in samples with tumor diameter >2.0 cm (*p* = 0.003) and higher TNM stage (*p* < 0.001). However, MCM3 intensity did not exhibit significant differences in terms of histological grade or lymph node metastasis (Table 2). More importantly, a positive correlation between ZMIZ2 and MCM3 was identified in the TNBC group (r = 0.791, *p* < 0.001) (Table 3), further confirming that ZMIZ2 could positively regulate MCM3. Furthermore, the Kaplan–Meier survival curves showed that the survival of patients with high MCM3 expression levels was shorter than that of patients with low MCM3 expression levels (Fig. 3C), illustrating that high MCM3 expression levels were linked to an unfavorable prognosis in patients with TNBC.

**Table 2:** The MCM3 expression in TNBC patients with different clinical parameters

Clinical features	N	MCM3 expression				χ^2^	*p*
		–	+	++	+++		
Tumor size						14.202	0.003
≤2 cm	66	11	21	20	14		
>2 cm	76	5	20	13	38		
Histological grade						6.496	0.370
1	44	3	9	12	20		
2	42	7	11	9	15		
3	56	6	21	12	17		
Lymph node metastasis						0.767	0.857
N = 0	50	6	16	12	16		
N > 0	92	10	25	21	36		
TNM stage						22.735	<0.001
I-IIb	51	6	26	4	15		
IIIa-IV	91	10	15	29	37		

Note: MCM3, minichromosome maintenance complex component 3; TNBC, Triple-negative breast cancer. The expression intensity of immunostaining was graded as: –, negative; +, weak; ++, moderate; and +++, strong.

**Table 3:** The expression intensity correlation between ZMIZ2 and MCM3 in TNBC and non-TNBC

Group	MCM3	ZMIZ2 expression				r	*p*
		–	+	++	+++		
NCBI	– (2)	0	2	0	0	0.791	<0.001
	+ (3)	2	1	0	0		
	++ (25)	0	6	19	0		
	+++ (45)	0	2	7	36		
Non-NCBI	– (14)	8	6	0	0	–0.137	0.270
	+ (38)	20	18	0	0		
	++ (8)	8	0	0	0		
	+++ (7)	4	3	0	0		

Note: ZMIZ2, zinc finger miz-type containing 2; MCM3, minichromosome maintenance complex component 3; TNBC, Triple-negative breast cancer. The expression intensity of immunostaining was graded as: –, negative; +, weak; ++, moderate; and +++, strong.

### 3.5 MCM3 Knockdown Reversed the Effects of ZMIZ2 Overexpression in TNBC Cells In Vitro

**Figure 4:**
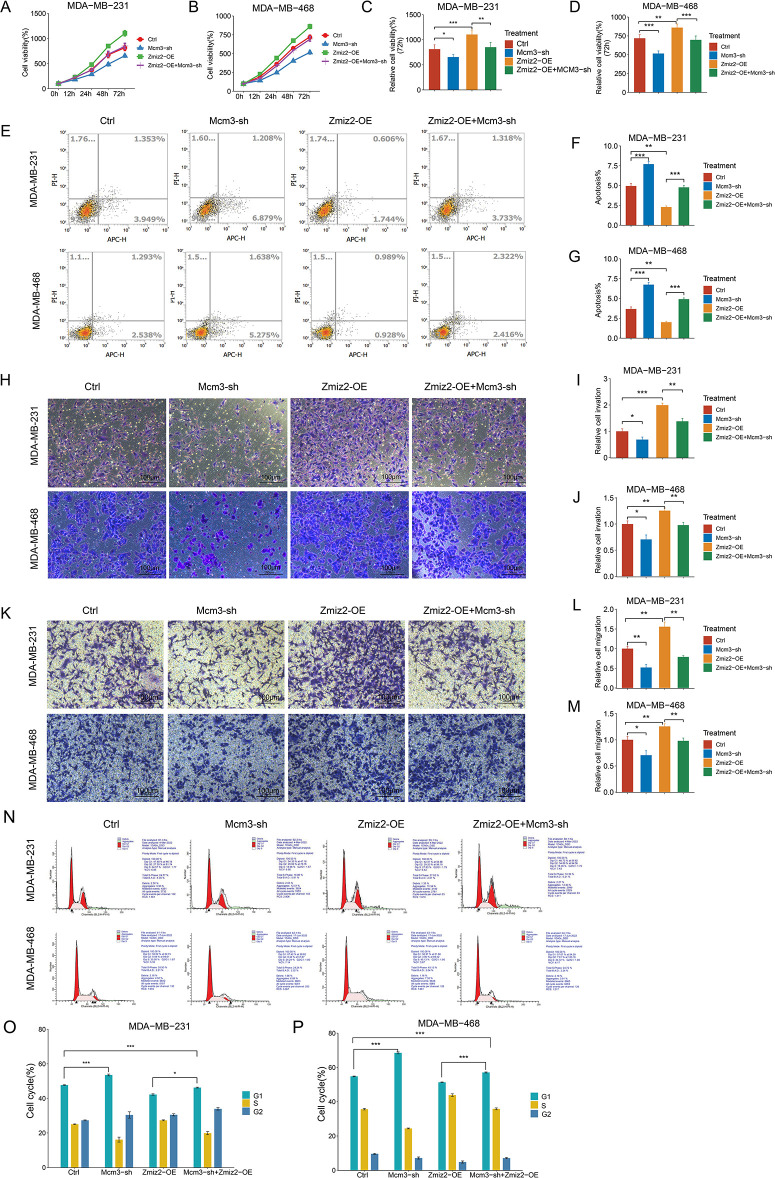
ZMIZ2 regulates cell proliferation, apoptosis, migration, and invasion through minichromosome maintenance complex component 3 (MCM3). MDA-MB-231 and MDA-MB-468 cells were transfected with MCM3-shRNA (MCM3-sh), ZMIZ2 overexpression plasmids (ZMIZ2-OE), and ZMIZ2-OE+MCM3-sh, respectively and cells that were transfected with the empty plasmid were used as control. (**A**–**D**): Cell Counting Kit-8 assay showing the cell viability of MDA-MB-231 and MDA-MB-468 cells during 72 h after different treatments. (**E**–**G**): Flow cytometry revealing the apoptosis of MDA-MB-231 and MDA-MB-468 cells after different treatments. (**H**–**M**): Transwell assays displaying the migration and invasion of MDA-MB-231 and MDA-MB-468 cells after different treatments. (**N**–**P**): Flow cytometry analyzed cell cycle of MDA-MB-231 and MDA-MB-468 cells after different treatments. *p < 0.05, **p < 0.01, and ***p < 0.001

To explore whether ZMIZ2 participates in TNBC progression through MCM3, we studied the effect of MCM3 knockdown on the malignant behavior of TNBC cells and explored whether MCM3 knockdown could reverse the function of ZMIZ2 overexpression in TNBC cells *in vitro*. The viability of MDA-MB-231 and MDA-MB-468 cells in the MCM3-sh group was clearly reduced relative to the control group, indicating that MCM3 knockdown inhibited TNBC cell proliferation (Fig. 4A–D). Moreover, cell viabilities of the ZMIZ2-OE+MCM3-sh group were observably decreased relative to those of the ZMIZ2-OE group, suggesting that the promoting effect of ZMIZ2 overexpression on TNBC cell proliferation was eliminated by MCM3 knockdown (Fig. 4A–D). The flow cytometry indicated that the cell apoptosis proportion was increased in the MCM3-sh group compared to that in the control group, and it was also elevated in the ZMIZ2-OE+MCM3-sh group compared to that in the ZMIZ2-OE group (Fig. 4E–G). Thus, MCM3 knockdown caused a significant increase in TNBC cell apoptosis, and the decreased proportion of apoptotic cells caused by ZMIZ2 overexpression was reversed by MCM3 knockdown. Transwell assays indicated that both cell migration and invasion increased after MCM3 knockdown alone, and the promoting effects of ZMIZ2 overexpression on cell migration and invasion were counteracted by the concurrent knockdown of MCM3 (Fig. 4H–M). The results of the cell cycle assay demonstrated an elevated proportion of cells in the G1 phase and a reduced proportion of cells in the G1 phase following MCM3 knockdown (Fig. 4N–P). Moreover, in the ZMIZ2-OE+MCM3-sh group, the trend change caused by the overexpression of ZMIZ2 was weakened after MCM3 knockdown (Fig. 4N–P). Thus, the oncogenic activity of ZMIZ2 in TNBC cells required MCM3 involvement.

### 3.6 MCM3 Knockdown Reversed the Function of ZMIZ2 Overexpression on TNBC Tumor Growth In Vivo

**Figure 5:**
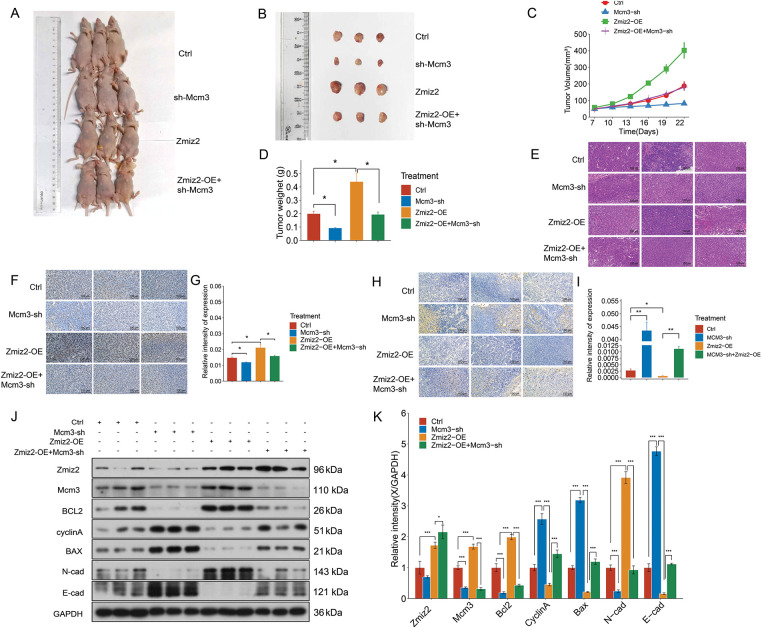
ZMIZ2 promoted tumor growth in triple-negative breast cancer xenografted mice. MDA-MB-231 cells, that were transfected with empty plasmid, MCM3-sh, ZMIZ2-OE, and ZMIZ2-OE+MCM3-sh, respectively, were subcutaneously injected into the groin of each mouse to establish xenograft models. (**A**): The representative images of subcutaneous tumors in nude mice from different groups. (**B**): The size of xenograft tumor of different groups. (**C**): The tumor volume changes of different groups. (**D**): The tumor weights of different groups. (**E**): Hematoxylin and eosin staining showed the pathological changes of tumor tissues in different groups. (**F**,**G**): Immunohistochemical staining showed the Ki67 expression in mouse subcutaneous tumor tissues. (**H**,**I**): TUNEL staining revealed the apoptosis is in the mouse subcutaneous tumor tissues. (**J**,**K**): Western blot assay determined the expression levels of epithelial–mesenchymal transition-, apoptosis-, and cell cycle-related markers in different groups. *p < 0.05, **p < 0.01, and ***p < 0.001

To further verify the relationship between ZMIZ2 and MCM3 *in vivo*, we established a mouse xenograft model and monitored tumor growth (Fig. 5A). After 3 weeks, the tumor weights were measured (Fig. 5B). The growth curve of xenografted TNBC tumors in mice showed that with an increase in the number of days, the tumor volume increased after ZMIZ2 overexpression but remarkably decreased after MCM3 knockdown (Fig. 5C). In addition, tumor weight showed similar changes; the tumor weight increased after ZMIZ2 overexpression, but remarkably decreased after MCM3 knockdown (Fig. 5D). Simultaneously, tumor growth in the ZMIZ2-OE+ CM3-sh group was significantly inhibited compared to that in the ZMIZ2-OE group (Fig. 5B–D). In addition, HE staining showed that ZMIZ2 overexpression increased nuclear division and relieved inflammatory cell infiltration in tumor tissues, whereas MCM3 knockdown caused evident interstitial fibrosis (Fig. 5E). To detect the proliferation and apoptosis of TNBC cells in the subcutaneous tumor tissues of mice after different treatments, IHC staining for Ki-67 and tunnel assays were performed. We observed that MCM3 silencing inhibited cell proliferation (Fig. 5F,G) and promoted apoptosis (Fig. 5H,I), whereas ZMIZ2 overexpression had opposite effects on cell proliferation and apoptosis (Fig. 5F–I). Moreover, co-transfection with ZMIZ2-OE and MCM3-sh largely eliminated the influence of ZMIZ2 overexpression on proliferation and apoptosis (Fig. 5F–I). To verify these findings, we measured the expression of markers related to apoptosis, proliferation, and metastasis. The expression levels of MCM3, BCL2, and N-cadherin increased, whereas those of cyclin A, BAX, and E-cadherin decreased in ZMIZ2-overexpressing tumor tissues (Fig. 5J,K). Changes in the expression of these markers were observed in MCM3-silenced tumor tissues. In addition, when MCM3 expression was suppressed in ZMIZ2-overexpressed mice, the expression of these markers caused by ZMIZ2overexpression was reversed (Fig. 5J,K). Overall, these data implied that ZMIZ2 contributed to TNBC tumor growth *in vivo* by regulating MCM3 expression.

### 3.7 Analysis of the Regulatory Mechanism of ZMIZ2/MCM3 Axis in TNBC Based on Our Transcriptome Sequencing Data

**Figure 6:**
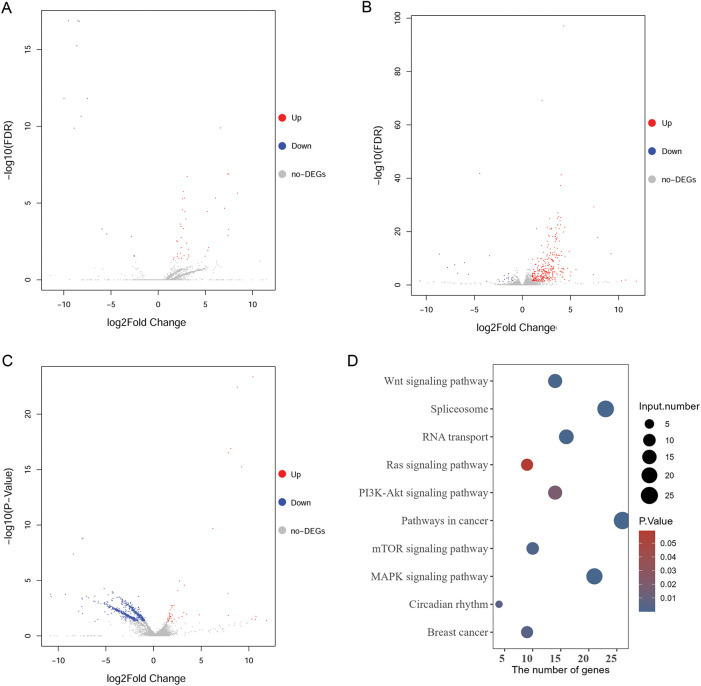
Analysis of DEGs and pathways based on our transcriptome sequencing analysis. (**A**): Volcano plot of DEGs between the MCM3-sh and Ctrl groups. (**B**): Volcano plot of DEGs between the ZMIZ2-OE and Ctrl groups. (**C**): Volcano plot of DEGs between the ZMIZ2-OE and MCM3-sh groups. (**D**): KEGG enrichment results for DEGs between the ZMIZ2-OE and MCM3-sh groups. DEGs, differentially expressed genes; KEGG, Kyoto Encyclopedia of Genes and Genomes; MCM3, minichromosome maintenance complex component 3

**Figure 7:**
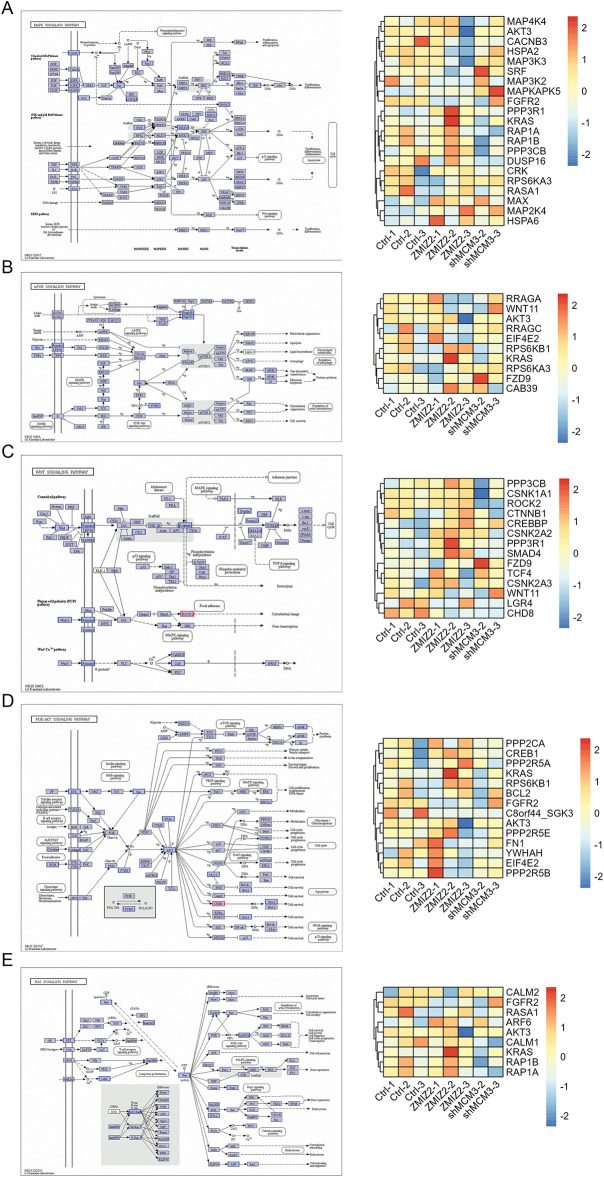
The KEGG pathway annotation map and expression heat map of pathway-related DEGs in different samples. (**A**): MAPK signaling pathway. (**B**): mTOR signaling pathway. (**C**): Wnt signaling pathway. (**D**): PI3K–AKT signaling pathway. (**E**): Ras signaling pathway. DEGs, differentially expressed genes; KEGG, Kyoto Encyclopedia of Genes and Genomes

To further elucidate the regulatory mechanism of the ZMIZ2/MCM3 axis in promoting TNBC, we performed RNA sequencing of tumor tissues from a xenograft mouse model in the control, MCM3-sh, and ZMIZ2-OE groups. A total of 52 DEGs were identified between the MCM3-sh and control groups (Fig. 6A), 370 DEGs were identified between the ZMIZ2-OE and control groups (Fig. 6B), and 682 DEGs were screened between the ZMIZ2-OE and MCM3-sh groups, including 46 upregulated and 636 downregulated genes (Fig. 6C). We then conducted a pathway enrichment analysis for the DEGs between the ZMIZ2-OE and MCM3-sh groups. These DEGs were significantly associated with multiple pathways (Table 4, Fig. 6D) such as the MAPK, mTOR, Wnt, PI3K/AKT, and Ras signaling pathways. The KEGG pathway annotation map and expression heat map of pathway-related DEGs in different samples are shown in Fig. 7A–E.

**Table 4:** The KEGG pathway enrichment results for DEGs between ZMIZ2-OE and MCM3-sh groups based on our transcriptome sequencing data

ID	Pathway definition	Gene number	Gene list
ko03040	Spliceosome	23	HSPA2, HSPA6, SF3B1, PRPF40B, DHX16, SRSF10, DHX15, SRSF5, SRSF7, SRSF6, SRSF1, SRSF2, HNRNPU, HNRNPK, HNRNPM, PCBP1, TRA2A, TRA2B, HNRNPA1L2, HNRNPA3, DDX5, RBM25, EIF4A3
ko03013	RNA transport	16	EIF1, SMN2, PABPC3, PNN, SUMO1, PABPC1, UPF2, EIF5, NUP153, FXR1, EIF4E2, EIF4G2, FMR1, NUP58, EIF4A3, EIF4A2
ko04010	MAPK signaling pathway	21	HSPA2, RPS6KA3, SRF, MAP4K4, PPP3R1, MAP3K2, MAP3K3, MAPKAPK5, MAX, KRAS, RAP1A, RAP1B, CACNB3, AKT3, MAP2K4, CRK, PPP3CB, DUSP16, FGFR2, RASA1, HSPA6
ko04310	Wnt signaling pathway	14	LGR4, CSNK1A1, PPP3R1, TCF4, SMAD4, FZD9, ROCK2, CTNNB1, CREBBP, CHD8, PPP3CB, CSNK2A3, CSNK2A2, WNT11
ko05200	Pathways in cancer	26	SMAD4, CTNNB1, FOXO1, AKT3, IFNGR2, CRK, RARA, CEBPA, NCOA1, CALM2, CALM1, FZD9, RPS6KB1, KRAS, TCF4, FGFR2, GSTT2, BDKRB2, BMP2|FN1, MAX, ROCK2, CREBBP, PTCH1, WNT11, BCL2
ko04150	mTOR signaling pathway	10	RPS6KA3, RRAGA, RRAGC, FZD9, CAB39, RPS6KB1, EIF4E2, AKT3, WNT11, KRAS
ko05224	Breast cancer	9	TCF4, NCOA1, CSNK1A1, FZD9, RPS6KB1, CTNNB1, AKT3, WNT11, KRAS
ko04710	Circadian rhythm	4	ARNTL, NR1D1, PRKAG2, CREB1
ko04151	PI3K-Akt signaling pathway	14	EIF4E2, FN1, PPP2CA, RPS6KB1, CREB1, PPP2R5A, YWHAH, AKT3, PPP2R5E, KRAS, FGFR2, C8orf44-SGK3, PPP2R5B, BCL2
ko04014	Ras signaling pathway	9	CALM2, CALM1, RAP1B, RAP1A, ARF6, AKT3, FGFR2, RASA1, KRAS

Note: KEGG, Kyoto Encyclopedia of Genes and Genomes; DEGs, differentially expressed genes; ZMIZ2, zinc finger miz-type containing 2; MCM3, minichromosome maintenance complex component 3; MAPK, mitogen-activated protein kinase; mTOR, mechanistic target of rapamycin; PI3K-Akt, phosphatidylinositol 3-kinase-protein kinase B.

### 3.8 Analysis of Key Pathways Associated with TNBC Based on TCGA Data

Based on TCGA data, we further analyzed DEGs and their enriched pathways in TNBC. Using this threshold value, we screened 3833 DEGs in the TNBC vs. normal group, including 1729 upregulated and 2104 downregulated genes. We then conducted a pathway enrichment analysis of the DEGs. Consistent with the analysis results of our transcriptome sequencing data, both the GSEA (Table 5) and KEGG pathway (Table 6) enrichment results showed that pathways, such as the MAPK, mTOR, Wnt, and Ras signaling pathways, were also significantly enriched in DEGs between TNBC and normal samples.

**Table 5:** The GSEA results of DEGs between TNBC and normal samples downloaded from TCGA database

ID	Pathway definition	NES	Size	*p*	Gene list
hsa04010	KEGG_MAPK_SIG NALING_PATHWAY	1.48	266	0.0516	CACNB3, IKBKB, RPS6KA1, NFKB2, MAPK13, MAPK7, MAPK8IP3, RELA, DAXX, PPP5C, TRAF2, TAB1
hsa04310	KEGG_WNT_SIG NALING_PATHWAY	1.46	150	0.0587	PPARD, VANGL2, CSNK1E, NKD2, DVL3, TCF7, AXIN1, CTBP2, FZD7
hsa04150	KEGG_MTOR_SIGN ALING_PATHWAY	1.36	50	0.119	TNF, PIK3CD, EIF4B, EIF4E, EIF4EBP1, AKT1, AKT2, VEGFD, PIK3R5
hsa04115	KEGG_P53_SIGN ALING_PATHWAY	1.90	66	0.012	TP53, PIDD1, CDK2, STEAP3, CD82, CASP9, TSC2, DDB2, PERP, SFN
hsa04110	KEGG_CELL_CYCLE	1.87	124	0.004	TP53, MCM3, HDAC1, FZR1, MCM5, CDK2, MCM7, ANAPC2, ANAPC7A, NAPC5, MCM2
hsa05200	KEGG_PATHWAYS _IN_CANCER	1.85	322	0.002	TP53, DVL2, TRAF4, PPARD, IKBK, ITGA3, CBLC, SMO, STK36, PIAS3
hsa03018	KEGG_RNA_DEG RADATION	1.79	56	0.005	CNOT3, EDC3, EDC4, EXOSC10, EXOSC2, SKIV2L, ENO2, PARN, CNOT10, DCP1B
hsa03030	KEGG_DNA_REP LICATION	1.74	36	0.009	LIG1, POLA2, MCM3, POLD1, MCM5, MCM7, POLE, MCM2, RFC2
hsa04210	KEGG_APOPTOSIS	1.71	86	0.089	TP53, IKBKB, DFFB, RELA, TRAF2, CAPN1, CASP9, TNFRSF10A, MAP3K14, CASP6, NFKB1

**Table 6:** The KEGG enrichment results of DEGs between TNBC and normal samples downloaded from TCGA database

ID	Pathway definition	Gene number	Gene list
hsa04110	Cell cycle	24	CDC7, E2F1, E2F2, E2F4, RBL1, SKP2, CHEK1, CDC20, ESPL1, MCM2, MCM3, MCM5, MCM6, CCNE1
hsa03030	DNA replication	10	POLD3, DNA2, POLD1, POLD2, RNASEH1, MCM2, POLA2, MCM3, MCM5, MCM6
hsa04010	MAPK signaling pathway	15	TRAF2, TNF, RELA, GNA12, RELB, MAP4K2, MKNK1, NFKB2, DUSP4, RPS6KA4, MAP3K14, MAP3K11
hsa04310	Wnt signaling pathway	6	PPARD, VANGL1, PRICKLE2, MMP7, FZD4, TCF7L1
hsa05200	Pathways in cancer	35	E2F1, BID, TRAF1, E2F2, TRAF2, E2F3, PPARD, ADCY7, MMP9, ARNT2, GNA12, PML, NFKBIA
hsa04150	mTOR signaling pathway	2	TNF, PIK3CD
hsa04210	Apoptosis	10	BID, TRAF2, TNF, RELA, PIK3CD, CSF2RB, NFKBIA, BIRC3, MAP3K14, BIRC2
hsa04660	T cell receptor signaling pathway	9	TNF, NFKBIE, RASGRP1, RELA, SOS1, NCK1, PIK3CD, NFKBIA, MAP3K14
hsa03013	RNA transport	12	NDC1, EIF4A3, EIF3B, NUP62, XPO5, PABPC4, ALYREF, POP1, NUP93, NUP85, NUPL2, TACC3
hsa04014	Ras signaling pathway	7	RAB5B, RASGRP1, RELA, SOS1, PIK3CD, GNG11, PDGFD

### 3.9 Ras/MAPK Signaling Pathway Participates in ZMIZ2/MCM3-Mediated TNBC Progression

**Figure 8:**
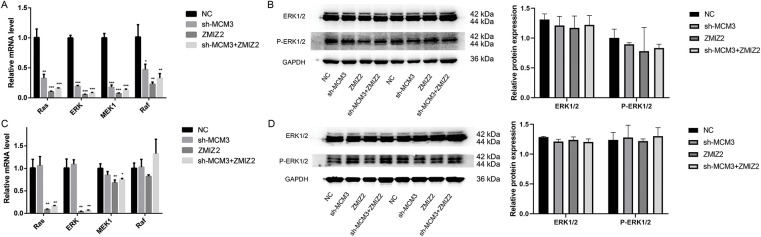
Effect of the ZMIZ2/MCM3 axis on Ras/MAPK signaling pathway *in vitro* and *in vivo*. (**A**): Quantitative reverse transcription polymerase chain reaction (qRT-PCR) showing the mRNA expressions of Ras, ERK, MEK1, and Raf in triple-negative breast cancer (TNBC) cells with different treatments. (**B**): Western blot revealing the protein expression of ERK1/2 and p-ERK1/2 in TNBC cells with different treatments. (**C**): qRT-PCR showing the protein expressions of ERK1/2 and p-ERK1/2 in xenografted tumors of different groups. (**D**): Western blot revealingtumor metastasis by inhibiting the the protein expression of ERK1/2 and p-ERK1/2 in xenografted tumors of different groups. *p < 0.05, **p < 0.01, and ***p < 0.001

The Ras/MAPK signaling pathway participates in cell growth, development, differentiation, and apoptosis and contributes to the development of many tumors. Therefore, we investigated the effect of the ZMIZ2/MCM3 axis on the Ras/MAPK signaling pathway *in vitro* and *in vivo*. ZMIZ2 overexpression and MCM3 knockdown reduced mRNA expression of Ras, ERK, MEK1, and Raf. Moreover, MCM3 knockdown largely eliminated the influence of ZMIZ2 overexpression on the expression of these genes (Fig. 8A). Although ERK1/2 and p-ERK1/2 protein levels were reduced after ZMIZ2 overexpression, the difference was not significant (Fig. 8B). *In vivo* experiments showed that the expression levels of Ras, ERK, MEK1, and Raf decreased after ZMIZ2 overexpression, which was partially reversed after MCM3 knockdown (Fig. 8C). No significant changes in ERK1/2 and p-ERK1/2 protein levels were observed after ZMIZ2 overexpression or MCM3 knockdown (Fig. 8D).

## 4 Discussion

**Figure 9:**
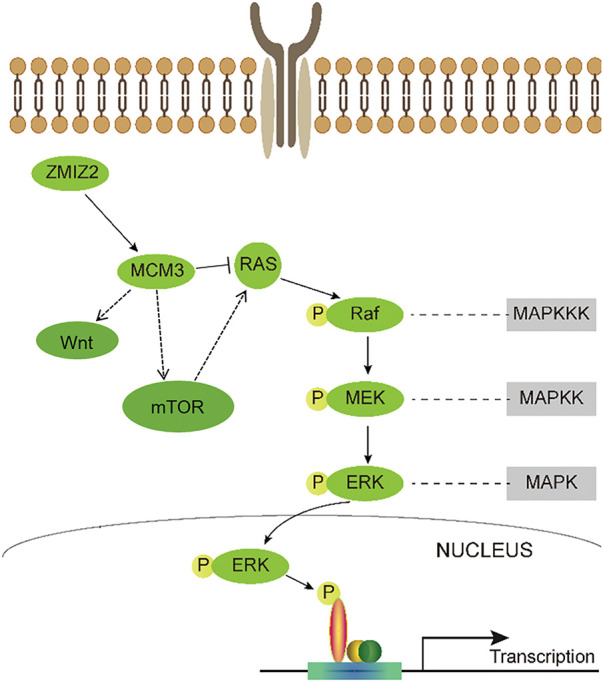
A schematic diagram summarizing the ZMIZ2/MCM3 axis and proposed downstream signaling pathways

TNBC is characterized by a heightened risk of distant metastasis and unfavorable prognostic outcomes. Owing to the absence of specific molecular targets, chemotherapy remains the primary treatment approach for TNBC in clinical practice [31]. Significant progress has been made in targeted therapies for various cancers, which has also led to improved outcomes in TNBC [32]. For instance, addition of the PARP inhibitor olaparib as adjuvant therapy can promote overall survival in patients with early TNBC harboring BRCA1 or BRCA2 germline mutations [33]. Another recently approved targeted drug for metastatic TNBC, sacituzumab govitecan-HZIY, has demonstrated improved survival [34]. However, the low efficacy and slow progress of current targeted therapies warrant new therapeutic targets for TNBC. Therefore, in-depth research on the crucial mechanisms underlying TNBC has significant implications for enhancing clinical outcomes. In this study, ZMIZ2 promoted TNBC development both *in vitro* and *in vivo* by positively regulating MCM3. Moreover, high ZMIZ2 or MCM3 expression levels were linked to the degree of malignancy and poor prognosis in patients with TNBC. Furthermore, key pathways, such as the Ras/MAPK, PI3K/AKT/mTOR, and Wnt signaling pathways, are identified as potential downstream mechanisms of the ZMIZ2/MCM3 axis in TNBC. A schematic diagram summarizing the ZMIZ2/MCM3 axis and proposed downstream signaling pathways is shown in Fig. 9.

ZMIZ2 is a PIAS-like protein, whose enhanced expression promotes tumor growth in multiple cancers, such as prostate cancer and CRC [20]. In CRC, ZMIZ2 facilitates deubiquitylation and stabilization of β-catenin by recruiting USP7, thereby contributing to tumorigenesis [20]. Gan et al. have demonstrated that ZMIZ2 promotes malignant development in lung cancer [35]. Consistent with these findings, we observed that ZMIZ2 promoted TNBC development both *in vitro* and *in vivo*. Thus, ZMIZ2 may play an oncogenic role in the development of TNBC.

Our previous findings revealed that ZMIZ2 promotes TNBC progression by interacting with multiple genes, including MCM3 [21]. As an enzyme essential for the initiation and elongation of DNA replication, MCM3 dysfunction is often associated with abnormal DNA replication and subsequent malignant cell proliferation [36,37]. Elevated MCM3 expression has been reported in, hepatocellular carcinoma [38,39], oral squamous cell carcinoma [40], and papillary thyroid carcinoma [15]. In this study, we also confirmed that direct interaction with and positive regulation of MCM3 and knockdown of MCM3 remarkably reversed the effects of ZMIZ2 overexpression *in vitro* and *in vivo*. Therefore, ZMIZ2 may promote TNBC progression by modulating MCM3 expression.

Moreover, we determined the expression of EMT-, apoptosis-, and cell cycle-related markers following ZMIZ2 overexpression. EMT is a key process in cancer cell metastasis [41]. In TNBC, abnormal activation of EMT is implicated in the onset of metastasis and aggressive progression of the disease [42,43]. Apoptosis is frequently altered in cancer cells, leading to malignancy and metastasis. TNBC cancer cells can modulate apoptosis by regulating anti-apoptotic and pro-apoptotic proteins [44]. The cell cycle is a key process that ensures accurate division of cells and monitors their integrity. Cell cycle disruption is crucial for cancer onset and progression [45]. Our results revealed that ZMIZ2 overexpression upregulated MCM3, BCL2, and N-cadherin, and downregulated BAX, E-cadherin, and cyclin A in TNBC cells. These data further confirm that ZMIZ2 promotes TNBC by regulating EMT, apoptosis, and cell cycle processes. Given the important role of ZMIZ2 in cancer biology, our findings offer novel perspectives on the molecular mechanisms underlying TNBC and pave the way for the development of innovative therapeutic approaches.

To further explore the potential mechanism of the ZMIZ2/MCM3 axis in promoting TNBC, we performed RNA sequencing of tumor tissues from a xenograft mouse model in the control, MCM3-sh, and ZMIZ2-OE groups. A total of 682 DEGs were in the ZMIZ2-OE group compared to that in the MCM3-sh group, and these DEGs were involved in multiple pathways, including the MAPK, mTOR, Wnt, and Ras signaling pathways. These pathways were significantly enriched in DEGs based on TCGA TNBC data. We hypothesized that these pathways may be potential mechanisms mediating the function of the ZMIZ2/MCM3 axis in TNBC. The PI3K/AKT/mTOR signaling pathway is responsible for cell survival, proliferation, and metabolism [46]. This signaling pathway is frequently activated in different subtypes of breast cancer [47], including TNBC [48]. Aberrant Wnt signaling is a hallmark of various cancers and participates in multiple cellular processes such as cell proliferation, cell fate, and stem cell pluripotency. Dysfunction of the Wnt signaling pathway has been observed in TNBC. Additionally, ZMIZ2 can positively regulate Wnt/β-catenin signaling pathway [49]. The promoting effects of the MAPK pathway have been demonstrated in various tumors [50]. Xu et al. have demonstrated that TSTA3 promotes esophageal cancer development by acting on MMP2 and MMP9 through the MAPK–ERK pathway [51]. Nevertheless, Peng et al. have reported that 20 (S)-protopanaxadiol restrained TNBC tumor metastasis by inhibiting the EGFR-mediated MAPK pathway [52]. The RAS/MAPK pathway is also a signaling pathway responsible for cell growth, proliferation, and differentiation. Activation of the RAS/MAPK pathway is prevalent in TNBC/basal-like cancers [53]. In the present study, these pathways were significantly enriched, supporting their role in mediating the functions of ZMIZ2 and MCM3 in TNBC. Nevertheless, our experiments showed that ZMIZ2 overexpression inhibited the Ras/MAPK signaling pathway, and knockdown of MCM3 eliminated the influence of ZMIZ2 overexpression on this pathway to a large extent. Therefore, we hypothesized that suppression of Ras/MAPK signaling by ZMIZ2 may be context-specific or potentially compensatory. The role of ZMIZ2 in regulating MAPK signaling may depend on various cellular factors, such as the tumor microenvironment [54] and stage of tumor progression, or it may be counterbalanced by the activation of other oncogenic pathways, such as the PI3K/AKT/mTOR pathway, which is known to cooperate with Ras/MAPK signaling in cancer [55]. Whether the ZMIZ2/MCM3 axis contributes to TNBC development by altering the RAS/MAPK signaling pathway remains to be verified and will be further studied by introducing MAPK/ERK inhibitors.

Our results showed that high ZMIZ2 and MCM3 expression levels were associated with the degree of malignancy of TNBC in clinical samples, highlighting their potential as therapeutic targets for TNBC. Both ZMIZ2 and MCM3 play significant roles in cell proliferation and survival, making them attractive candidates for therapeutic interventions. ZMIZ2, a downstream target of SGK3, stabilizes β-catenin and promotes tumor progression, which has been linked to poor prognosis in estrogen receptor-positive breast cancer [56]. Although the role of ZMIZ2 in TNBC is yet to be fully explored, its involvement in key signaling pathways suggests that inhibiting its function could interfere with tumor growth and metastasis, offering a potential therapeutic strategy. MCM3 is essential for DNA replication and cell-cycle regulation [57]. Previous studies have shown that cancer cells experiencing chronic replication stress display dysfunctional DNA replication and impaired DNA damage response, offering potential therapeutic vulnerability in tumors. DNA replication proteins play key roles in tumorigenesis and progression, highlighting their potential as valuable therapeutic targets in clinical settings [58]. Therefore, targeting MCM3 may hinder DNA replication in rapidly proliferating tumor cells, including those in TNBC, and MCM3 may be a promising candidate for drug development. Further research is required to identify small molecules or biologics capable of specifically targeting ZMIZ2 or MCM3 with high efficacy and minimal toxicity.

This study has some limitations. First, the effect of ZMIZ2 on MCM3 was not explored using sh-ZMIZ2 data *in vivo*. Further studies including more *in vivo* functional experiments should be performed to clarify the regulatory role of MIZ2 on MCM3 in TNBC development. Second, the regulatory mechanisms of the ZMIZ2/MCM3 axis on the activation of key pathways, such as the Ras/MAPK, PI3K/AKT/mTOR, and Wnt signaling pathways, have not been studied in depth by introducing inhibitors targeting these pathways. Finally, we conducted a transcriptome analysis to explore the DEGs associated with ZMIZ2. The top DEGs that may mediate TNBC proliferation or metastasis were neither experimentally validated nor compared with the findings from TCGA data. Further research with additional functional experiments is needed to explore the role and possible mechanisms of the ZMIZ2/MCM3 axis in TNBC development.

In conclusion, ZMIZ2 expression was elevated in TNBC and promoted the development by positively regulating MCM3. Key pathways, such as the Ras/MAPK, PI3K/AKT/mTOR, and Wnt signaling pathways, may be key downstream mechanisms of the ZMIZ2/MCM3 axis in TNBC. This discovery provides a theoretical basis and promising direction for targeted diagnosis and treatment of TNBC.

## Data Availability

The data that support the findings of this study are available from the corresponding authors upon request.

## References

[1] Pandiselvi A, Rajagopal K. Causes, risk factors, and prevention of breast cancer: a comprehensive review. Lat Am J Pharm. 2023;42(2):559–65.

[2] Zhang Z, Zhang R, Li D. Molecular biology mechanisms and emerging therapeutics of triple-negative breast cancer. Biol Targets Ther. 2023;17:113–28. doi:10.2147/btt.s426392; 37767463 PMC10520847

[3] Bianchini G, De Angelis C, Licata L, Gianni L. Treatment landscape of triple-negative breast cancer—expanded options, evolving needs. Nat Rev Clin Oncol. 2022;19(2):91–113. doi:10.1038/s41571-021-00565-2; 34754128

[4] Xiao Y, Ma D, Yang YS, Yang F, Ding JH, Gong Y, et al. Comprehensive metabolomics expands precision medicine for triple-negative breast cancer. Cell Res. 2022;32(5):477–90. doi:10.1038/s41422-022-00614-0; 35105939 PMC9061756

[5] Bauer KR, Brown M, Cress RD, Parise CA, Caggiano V. Descriptive analysis of estrogen receptor (ER)-negative, progesterone receptor (PR)-negative, and HER2-negative invasive breast cancer, the so-called triple-negative phenotype: a population-based study from the California cancer Registry. Cancer. 2007;109(9):1721–8. doi:10.1002/cncr.22618; 17387718

[6] Dent R, Trudeau M, Pritchard KI, Hanna WM, Kahn HK, Sawka CA, et al. Triple-negative breast cancer: clinical features and patterns of recurrence. Clin Cancer Res. 2007;13(15):4429–34. doi:10.1158/1078-0432.ccr-06-3045; 17671126

[7] Bou Zerdan M, Ghorayeb T, Saliba F, Allam S, Bou Zerdan M, Yaghi M, et al. Triple negative breast cancer: updates on classification and treatment in 2021. Cancers. 2022;14(5):1253. doi:10.3390/cancers14051253; 35267561 PMC8909187

[8] Sedlackova H, Rask MB, Gupta R, Choudhary C, Somyajit K, Lukas J. Equilibrium between nascent and parental MCM proteins protects replicating genomes. Nature. 2020;587(7833):297–302. doi:10.1038/s41586-020-2842-3; 33087936

[9] Remus D, Beuron F, Tolun G, Griffith JD, Morris EP, Diffley JF. Concerted loading of Mcm2-7 double hexamers around DNA during DNA replication origin licensing. Cell. 2009;139(4):719–30. doi:10.1016/j.cell.2009.10.015; 19896182 PMC2804858

[10] Miller TC, Locke J, Greiwe JF, Diffley JF, Costa A. Mechanism of head-to-head MCM double-hexamer formation revealed by cryo-EM. Nature. 2019;575(7784):704–10. doi:10.1038/s41586-019-1768-0; 31748745 PMC6887548

[11] Woodward AM, Göhler T, Luciani MG, Oehlmann M, Ge X, Gartner A, et al. Excess Mcm2-7 license dormant origins of replication that can be used under conditions of replicative stress. J Cell Biol. 2006;173(5):673–83. doi:10.1083/jcb.200602108; 16754955 PMC2063885

[12] Ge XQ, Jackson DA, Blow JJ. Dormant origins licensed by excess Mcm2-7 are required for human cells to survive replicative stress. Genes Dev. 2007;21(24):3331–41. doi:10.1101/gad.457807; 18079179 PMC2113033

[13] Tye BK. MCM proteins in DNA replication. Annu Rev Biochem. 1999;68(1):649–86. doi:10.1146/annurev.biochem.68.1.649; 10872463

[14] Cao L, Zhao Y, Liang Z, Yang J, Wang J, Tian S, et al. Corrigendum: systematic analysis of MCM3 in pediatric medulloblastoma via multi-omics analysis. Front Mol Biosci. 2022;9:1076243. doi:10.3389/fmolb.2022.1076243; 36452454 PMC9703341

[15] Igci Y, Erkilic S, Igci M, Arslan A. MCM3 protein expression in follicular and classical variants of papillary thyroid carcinoma. Pathol Oncol Res. 2013;20(1):87–91. doi:10.1007/s12253-013-9662-9; 23821456

[16] Yang Q, Xie B, Tang H, Meng W, Jia C, Zhang X, et al. Minichromosome maintenance 3 promotes hepatocellular carcinoma radioresistance by activating the NF-κB pathway. J Exp Clin Cancer Res. 2019;38(1):263. doi:10.1186/s13046-019-1338-1; 31208444 PMC6580494

[17] Kato K, Toki T, Shimizu M, Shiozawa T, Fujii S, Nikaido T, et al. Expression of replication-licensing factors MCM2 and MCM3 in normal, hyperplastic, and carcinomatous endometrium: correlation with expression of Ki-67 and estrogen and progesterone receptors. Int J Gynecol Pathol. 2003;22(4):334–40. doi:10.1097/01.pgp.0000092129.10100.5e; 14501812

[18] Zhao Y, Wang Y, Zhu F, Zhang J, Ma X, Zhang D. Gene expression profiling revealed MCM3 to be a better marker than Ki67 in prognosis of invasive ductal breast carcinoma patients. Clin Exp Med. 2020;20(2):249–59. doi:10.1007/s10238-019-00604-4; 31980982

[19] Huang CY, Beliakoff J, Li X, Lee J, Li X, Sharma M, et al. hZimp7, a novel PIAS-like protein, enhances androgen receptor-mediated transcription and interacts with SWI/SNF-like BAF complexes. Mol Endocrinol. 2005;19(12):2915–29. doi:10.1210/me.2005-0097; 16051670

[20] Zhu Y, Gu L, Lin X, Cui K, Liu C, Lu B, et al. LINC00265 promotes colorectal tumorigenesis via ZMIZ2 and USP7-mediated stabilization of β-catenin. Cell Death Differ. 2020;27(4):1316–27. doi:10.1038/s41418-019-0417-3; 31527801 PMC7206056

[21] Zou X, Liu Y, Di J, Wei W, Watanabe N, Li J, et al. ZMIZ2 promotes the development of triple-receptor negative breast cancer. Cancer Cell Int. 2022;22(1):1–16. doi:10.1186/s12935-021-02393-x; 35101047 PMC8802436

[22] Masliah-Planchon J, Garinet S, Pasmant E. RAS-MAPK pathway epigenetic activation in cancer: miRNAs in action. Oncotarget. 2015;7(25):38892. doi:10.18632/oncotarget.6476; 26646588 PMC5122439

[23] Wang XF, Zhou QM, Du J, Zhang H, Lu YY, Su SB. Baicalin suppresses migration, invasion and metastasis of breast cancer via p38MAPK signaling pathway. Anti-Cancer Agents Med Chem. 2013;13(6):923–31. doi:10.2174/18715206113139990143; 23387975

[24] Meng X, Cai C, Wu J, Cai S, Ye C, Chen H, et al. TRPM7 mediates breast cancer cell migration and invasion through the MAPK pathway. Cancer Lett. 2013;333(1):96–102. doi:10.1016/j.canlet.2013.01.031; 23353055

[25] Hashimoto K, Tsuda H, Koizumi F, Shimizu C, Yonemori K, Ando M, et al. Activated PI3K/AKT and MAPK pathways are potential good prognostic markers in node-positive, triple-negative breast cancer. Ann Oncol. 2014;25(10):1973–9. doi:10.1093/annonc/mdu247; 25009009

[26] Wu N, Zhang J, Zhao J, Mu K, Zhang J, Jin Z, et al. Precision medicine based on tumorigenic signaling pathways for triple-negative breast cancer. Oncol Lett. 2018;16(4):4984–96; 30250564 10.3892/ol.2018.9290 PMC6144355

[27] Anders S, Pyl PT, Huber W. HTSeq—a python framework to work with high-throughput sequencing data. Bioinformatics. 2015;31(2):166–9. doi:10.1101/002824. 25260700 PMC4287950

[28] Love MI, Huber W, Anders S. Moderated estimation of fold change and dispersion for RNA-seq data with DESeq2. Genome Biol. 2014;15(12):1–21. doi:10.1186/s13059-014-0550-8; 25516281 PMC4302049

[29] Yu G, Wang LG, Han Y, He QY. clusterProfiler: an R package for comparing biological themes among gene clusters. Omics A J Integr Biol. 2012;16(5):284–7. doi:10.1089/omi.2011.0118; 22455463 PMC3339379

[30] Smyth GK. Limma: linear models for microarray data. In: Bioinformatics and computational biology solutions using R and Bioconductor. New York, NY, USA: Springer; 2005. p. 397–420.

[31] So JY, Ohm J, Lipkowitz S, Yang L. Triple negative breast cancer (TNBC): non-genetic tumor heterogeneity and immune microenvironment: emerging treatment options. Pharmacol Ther. 2022;237(4):108253. doi:10.1016/j.pharmthera.2022.108253; 35872332 PMC9378710

[32] Tong CW, Wu M, Cho W, To KK. Recent advances in the treatment of breast cancer. Front Oncol. 2018;8:227. doi:10.3389/fonc.2018.00227; 29963498 PMC6010518

[33] Tutt AN, Garber JE, Kaufman B, Viale G, Fumagalli D, Rastogi P, et al. Adjuvant olaparib for patients with BRCA1-or BRCA2-mutated breast cancer. N Engl J Med. 2021;384(25):2394–405. doi:10.1056/NEJMoa2105215; 34081848 PMC9126186

[34] Bardia A. New data for sacituzumab govitecan-hziy in the treatment of metastatic triple-negative breast cancer. Clin Adv Hematol Oncol. 2021;19(11):723–5. doi:10.1016/s1470-2045(19)30074-9; 34807017

[35] Gan X, Feng Y, Liu Y, Lin X, Yu X, Rong X, et al. Identification of zinc finger MIZ-type containing 2 as an oncoprotein enhancing NAD-dependent protein deacetylase sirtuin-1 deacetylase activity to regulate Wnt and Hippo pathways in non-small-cell lung cancer. Cell Mol Biol Lett. 2024;29(1):122. doi:10.1186/s11658-024-00636-z; 39266996 PMC11391738

[36] Ishimi Y. Regulation of MCM2-7 function. Genes Genet Syst. 2018;93(4):125–33. doi:10.1266/ggs.18-00026; 30369561

[37] Gao Z, Man X, Li Z, Bi J, Liu X, Li Z, et al. Correction: pLK1 promotes proliferation and suppresses apoptosis of renal cell carcinoma cells by phosphorylating MCM3. Cancer Gene Ther. 2022;29(5):627–7. doi:10.1038/s41417-022-00458-1; 35361959

[38] Zhuang L, Yang Z, Meng Z. Upregulation of BUB1B, CCNB1, CDC7, CDC20, and MCM3 in tumor tissues predicted worse overall survival and disease-free survival in hepatocellular carcinoma patients. BioMed Res Int. 2018;2018(1):7897346. doi:10.1155/2018/7897346; 30363964 PMC6186344

[39] Yang WX, Pan YY, You CG. CDK1, CCNB1, CDC20, BUB1, MAD2L1, MCM3, BUB1B, MCM2, and RFC4 may be potential therapeutic targets for hepatocellular carcinoma using integrated bioinformatic analysis. BioMed Res Int. 2019;2019(1):1245072. doi:10.1155/2019/1245072; 31737652 PMC6815605

[40] Rezazadeh F, Ebrahimi R, Andisheh-Tadbir A, Ashraf MJ, Khademi B. Evaluation of the Ki-67 and MCM3 expression in cytologic smear of oral squamous cell carcinoma. J Dent. 2017;18(3):207–11. PMC5634361 29034276

[41] Jung YY, Chinnathambi A, Alahmadi TA, Alharbi SA, Kumar AP, Sethi G, et al. Fangchinoline targets epithelial-mesenchymal transition process by modulating activation of multiple cell-signaling pathways. J Cell Biochem. 2022;123(7):1222–36. doi:10.1002/jcb.30279; 35621239

[42] Zeng Q, Li W, Lu D, Wu Z, Duan H, Luo Y, et al. CD146, an epithelial-mesenchymal transition inducer, is associated with triple-negative breast cancer. Proc Natl Acad Sci U S A. 2012;109(4):1127–32. doi:10.1073/pnas.1111053108; 22210108 PMC3268312

[43] Kashyap A, Umar S, Dev JRA, Prasad C. Dihydrotanshinone-I modulates epithelial mesenchymal transition (EMT) thereby impairing migration and clonogenicity of triple negative breast cancer cells. Asian Pac J Cancer Prev. 2021;22(7):2177. doi:10.31557/apjcp.2021.22.7.2177; 34319041 PMC8607078

[44] Messeha SS, Zarmouh NO, Mendonca P, Alwagdani H, Cotton C, Soliman KF. Effects of gossypol on apoptosis-related gene expression in racially distinct triple-negative breast cancer cells. Oncol Rep. 2019;42(2):467–78; 31173249 10.3892/or.2019.7179 PMC6610046

[45] Stewart ZA, Westfall MD, Pietenpol JA. Cell-cycle dysregulation and anticancer therapy. Trends Pharmacol Sci. 2003;24(3):139–45. doi:10.1016/s0165-6147(03)00026-9; 12628359

[46] Sharma VR, Gupta GK, Sharma AK, Batra N, Sharma DK, Joshi A, et al. PI3K/Akt/mTOR intracellular pathway and breast cancer: factors, mechanism and regulation. Curr Pharm Des. 2017;23(11):1633–8. doi:10.2174/1381612823666161116125218; 27848885

[47] Zhu K, Wu Y, He P, Fan Y, Zhong X, Zheng H, et al. PI3K/AKT/mTOR-targeted therapy for breast cancer. Cells. 2022;11(16):2508. doi:10.3390/cells11162508; 36010585 PMC9406657

[48] Costa RL, Han HS, Gradishar WJ. Targeting the PI3K/AKT/mTOR pathway in triple-negative breast cancer: a review. Breast Cancer Res Treat. 2018;169(3):397–406. doi:10.1007/s10549-018-4697-y; 29417298

[49] Lee SH, Zhu C, Peng Y, Johnson DT, Lehmann L, Sun Z. Identification of a novel role of ZMIZ2 protein in regulating the activity of the Wnt/β-catenin signaling pathway. J Biol Chem. 2013;288(50):35913–24. doi:10.1074/jbc.m113.529727; 24174533 PMC3861641

[50] Kamiyama M, Naguro I, Ichijo H. Functional analysis of apoptosis signal-regulating kinase family in a murine model of tumor metastasis. Yakugaku Zasshi. 2019;139(5):743–51. doi:10.1248/yakushi.18-00185-2; 31061344

[51] Xu EW, Yang J, Zhang L. TSTA3 gene promotes esophageal cancer invasion through MAPK-ERK pathway and downstream MMP2/9. Zhonghua Bing Li Xue Za Zhi. 2022;51(1):50–2; 34979755 10.3760/cma.j.cn112151-20210720-00519

[52] Peng B, He R, Xu Q, Yang Y, Hu Q, Hou H, et al. Ginsenoside 20(S)-protopanaxadiol inhibits triple-negative breast cancer metastasis *in vivo* by targeting EGFR-mediated MAPK pathway. Pharmacol Res. 2019;142(2):1–13. doi:10.1016/j.phrs.2019.02.003; 30735802

[53] Galiè M. RAS as supporting actor in breast cancer. Front Oncol. 2019;9:1199. doi:10.3389/fonc.2019.01199; 31781501 PMC6861383

[54] Liu Y, Xie B, Chen Q. RAS signaling and immune cells: a sinister crosstalk in the tumor microenvironment. J Transl Med. 2023;21(1):595. doi:10.1186/s12967-023-04486-9; 37670322 PMC10481548

[55] Li Q, Li Z, Luo T, Shi H. Targeting the PI3K/AKT/mTOR and RAF/MEK/ERK pathways for cancer therapy. Mol Biomed. 2022;3(1):47. doi:10.1186/s43556-022-00110-2; 36539659 PMC9768098

[56] Yuan L, Cai Y, Wang G, Liu X, Chen B, Zhou D, et al. SGK3 promotes estrogen receptor-positive breast cancer proliferation by activating STAT3/ZMIZ2 pathway to stabilise β-catenin. Br J Pharmacol. 2025;182(8):1856–75. doi:10.1111/bph.17453; 39876548

[57] Chen Y, Li LY, Li JD, He RQ, Huang ZG, Huang WY, et al. Expression, potential biological behaviour and clinical significance of MCM3 in pancreatic adenocarcinoma: a comprehensive study integrating high throughput sequencing, CRISPR screening and in-house immunohistochemistry. Ann Med. 2024;56(1):2405879. doi:10.1080/07853890.2024.2405879; 39310930 PMC11421141

[58] Song HY, Shen R, Mahasin H, Guo YN, Wang DG. DNA replication: mechanisms and therapeutic interventions for diseases. MedComm. 2023;4(1):e210. doi:10.1002/mco2.210; 36776764 PMC9899494

